# Supplementary material for: Runx2 and Runx3 differentially regulate articular chondrocytes during surgically induced osteoarthritis development
Source: Nat Commun. 2022 Oct 19;13:6187. doi: 10.1038/s41467-022-33744-5 (PMC9581901; doi:10.1038/s41467-022-33744-5)
Supplement: Supplementary file 1 — Supplementary Information [file 41467_2022_33744_MOESM1_ESM.pdf]

Runx2 and Runx3 differentially regulate articular chondrocytes during osteoarthritis development

Supplementary information

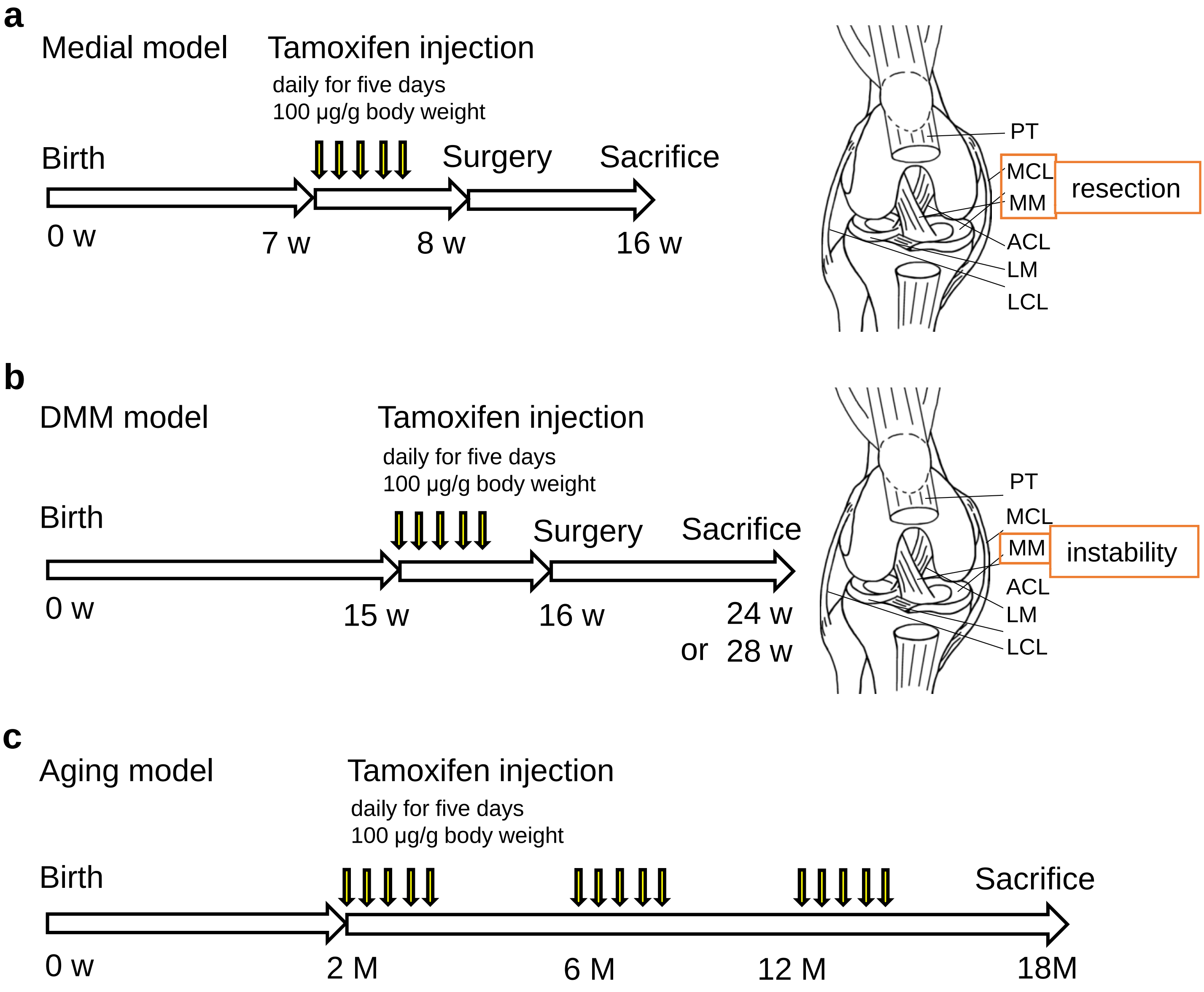

Supplementary Fig. 1 Schematic time course of osteoarthritis (OA) models. **a** Surgical model induced by resecting the medial collateral ligament and medial meniscus (medial model). **b** Surgical model induced by destabilizing the medial meniscus (DMM model). *Runx3* and *Runx2* knockout mice were sacrificed eight weeks and twelve weeks after surgery, respectively. **c** Natural course with aging (aging model). PT, patellar tendon; MCL, medial collateral ligament; MM, medial meniscus; ACL, anterior cruciate ligament; LM, lateral meniscus; LCL, lateral collateral ligament.

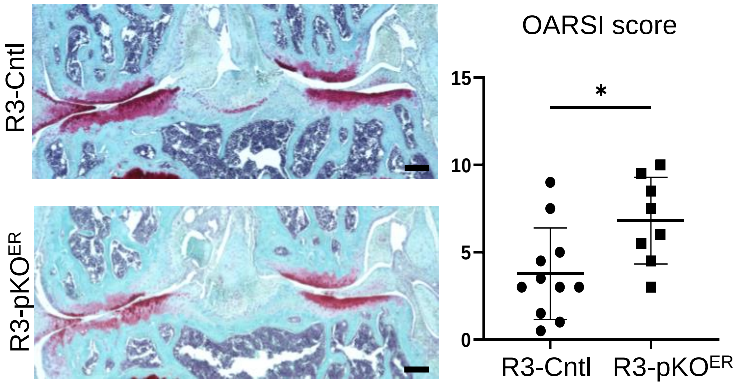

Supplementary Fig. 2 Development of OA in *Runx3*$^{fl/fl}$ (R3-Cntl) and *Prg4-Cre*^{ERT2};*Runx3*$^{fl/fl}$ (R3-pKO^{ER}) mice by destabilization of the medial meniscus (DMM model) at the age of 16 weeks. Tamoxifen injection was performed one week before surgery. The mice were sacrificed 8 weeks after the surgery. Scale bars indicate 200 µm. Experimental groups consisted of n = 11 and 8, respectively. OARSI scores are expressed as dot plots and means ± standard deviation (S.D.). **P* < 0.05, two-tailed Mann-Whitney U test.

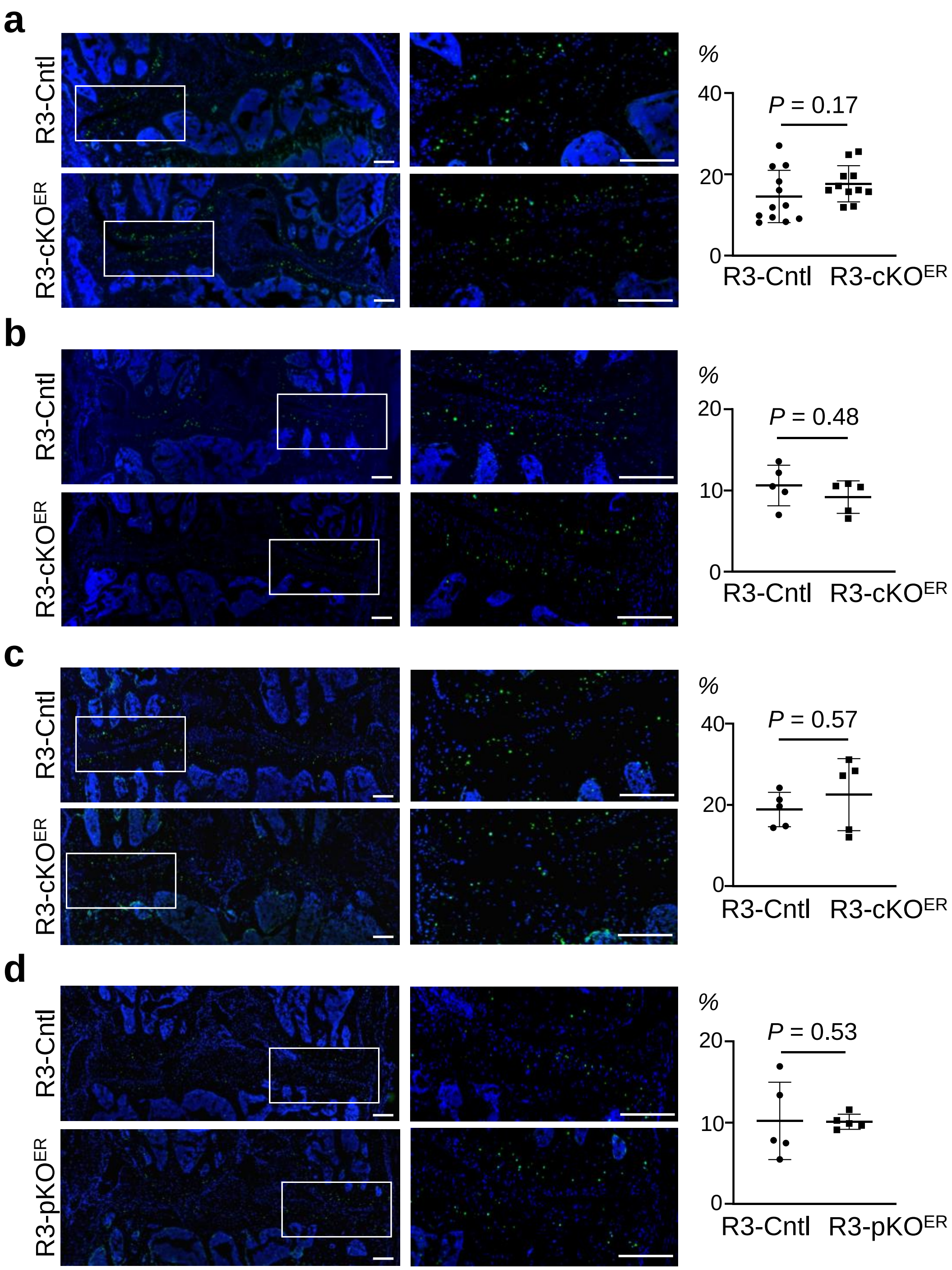

Supplementary Fig. 3 Chondrocyte apoptosis in articular cartilage of *Runx3*$^{fl/fl}$ (R3-Cntl) and *Col2a1-Cre*^{ERT2};*Runx3*$^{fl/fl}$ (R3-cKO^{ER}) or *Prg4-Cre*^{ERT2};*Runx3*$^{fl/fl}$ (R3-pKO^{ER}) mice. TUNEL staining of operated (**a**) and sham-operated (**b**) knee joints of 16-week-old R3-Cntl and R3-cKO^{ER} mice. Tamoxifen was injected at seven weeks, and the medial model surgery was performed at eight weeks. **c** TUNEL staining of knee joints of 18-month-old R3-Cntl and R3-cKO^{ER} mice, into which Tamoxifen was injected at 8 weeks, 6, and 12 months. **d** TUNEL staining of sham-operated knee joints of 16-week-old R3-Cntl and R3-pKO^{ER} mice, into which Tamoxifen was injected at seven weeks. Inset boxes in the low magnification images indicate regions of the high magnification images. Scale bars, 200 µm. Experimental groups consisted of n = 12 vs. 11 (**a**), or 5 (**b**, **c**, **d**), respectively. Rates of TUNEL-positive cells in the Safranin O-positive area are shown in the right panels. All data are expressed as dot plots and means ± S.D. All *P* values were over 0.05 in two-tailed Mann-Whitney U test.

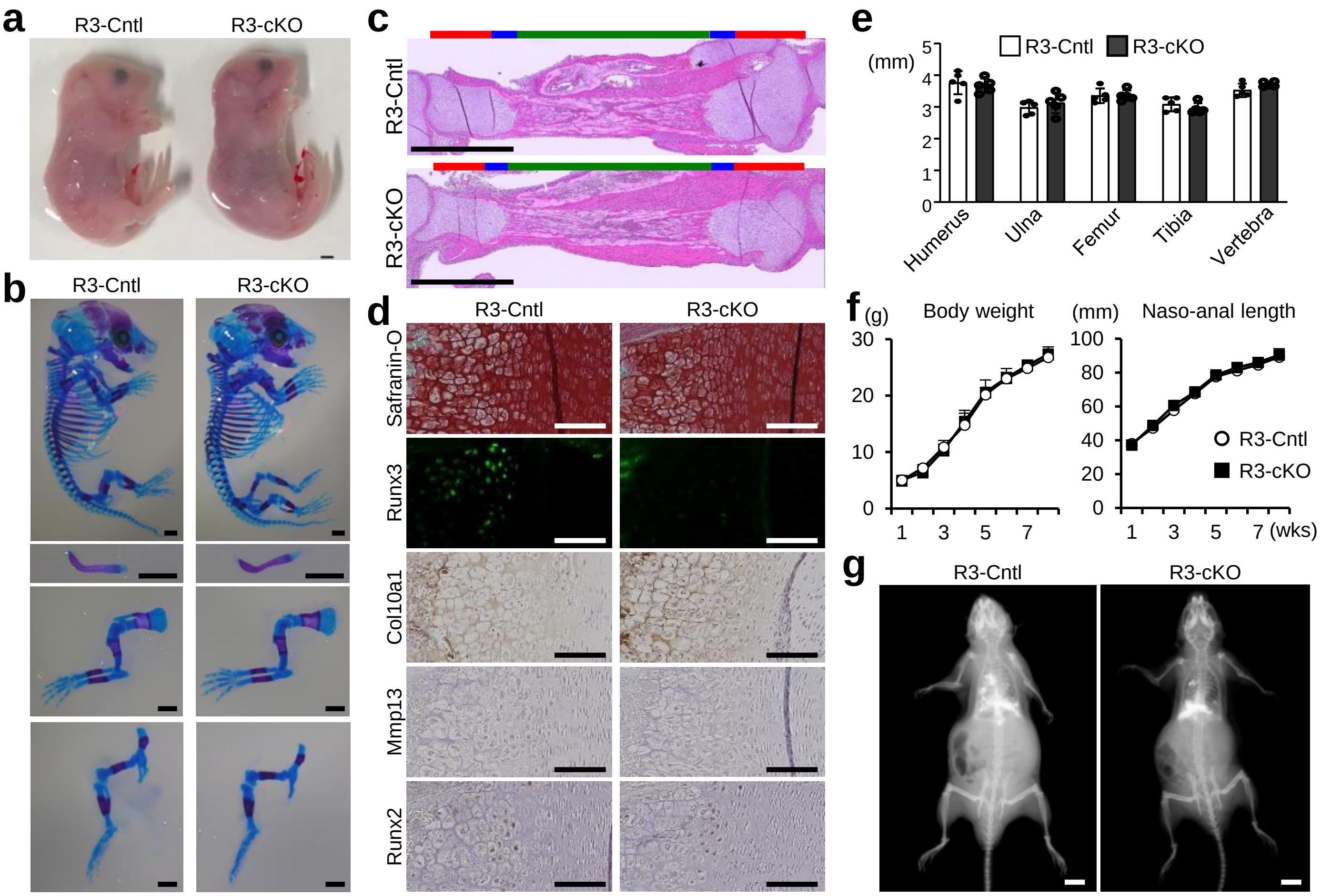

Supplementary Fig. 4 Skeletal formation in *Col2a1-Cre;Runx3$^{fl/fl}$* mice. **a** Gross appearance of *Runx3$^{fl/fl}$* (R3-Cntl) and *Col2a1-Cre;Runx3$^{fl/fl}$* (R3-cKO) littermate E18.5 embryos. Scale bars indicate 1 mm. **b** Double staining with Alizarin red and Alcian blue of the whole skeleton, clavicles, upper extremities, and lower extremities of R3-Cntl and R3-cKO littermate E18.5 embryos. Scale bars indicate 1 mm. **c** H&E staining of whole tibias ofR3- Cntl andR3- cKO littermate E18.5 embryos. Scale bars indicate 1 mm. Upper bars indicate lengths of the proliferative zone (red), hypertrophic zone (blue), and bone area (green). **d** Safranin O staining and immunohistochemistry of Runx3, Col10a1, Mmp13, and Runx2 in proximal tibias of R3-Cntl and R3-cKO littermate E18.5 embryos. Scale bars indicate 200 μm. Representative images among five mice in each genotype were shown.(**c**, **d**). **e** Length of long bones and vertebra (first to fifth lumbar spines) of R3-Cntl and R3-cKO littermate E18.5 embryos. Data are expressed as means ± S.D. of five mice per group. **f** Body weight and naso-anal length of R3-Cntl and R3-cKO littermates from 1- to 8-week-old. Data are expressed as means ± S.D. of R3-Cntl (n = 5) and R3-cKO mice (n = 7). **g** Plain radiographs of the entire bodies of R3-Cntl and R3-cKO littermates at 16 weeks. Scale bars indicate 1 cm.

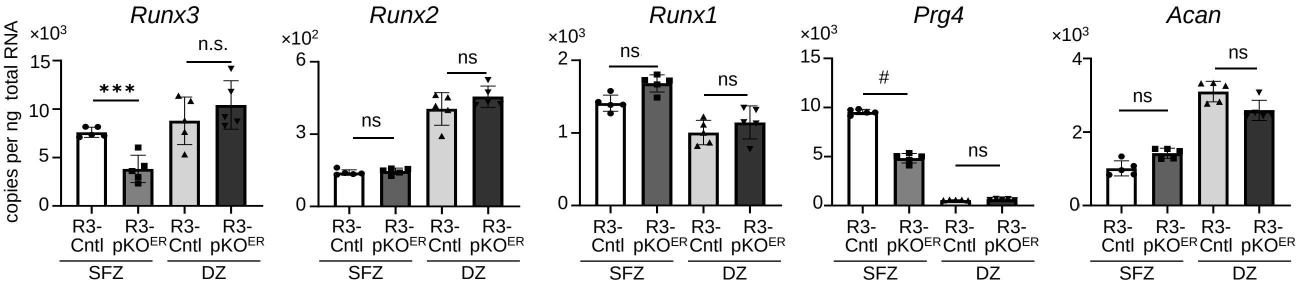

Supplementary Fig. 5 mRNA levels of Runx family members, *Prg4*, and *Acan* in primary SFZ and DZ chondrocytes obtained from *Runx3$^{fl/fl}$* (R3-Cntl) and *Prg4-Cre^{ERT2};Runx3$^{fl/fl}$* (R3-pKO^{ER}) mice. Data are expressed as means ± S.D. of five mice per group. ****P* < 0.001, #*P* < 0.0001, n.s., not significant; ordinary one-way ANOVA test with Turkey's post hoc test.

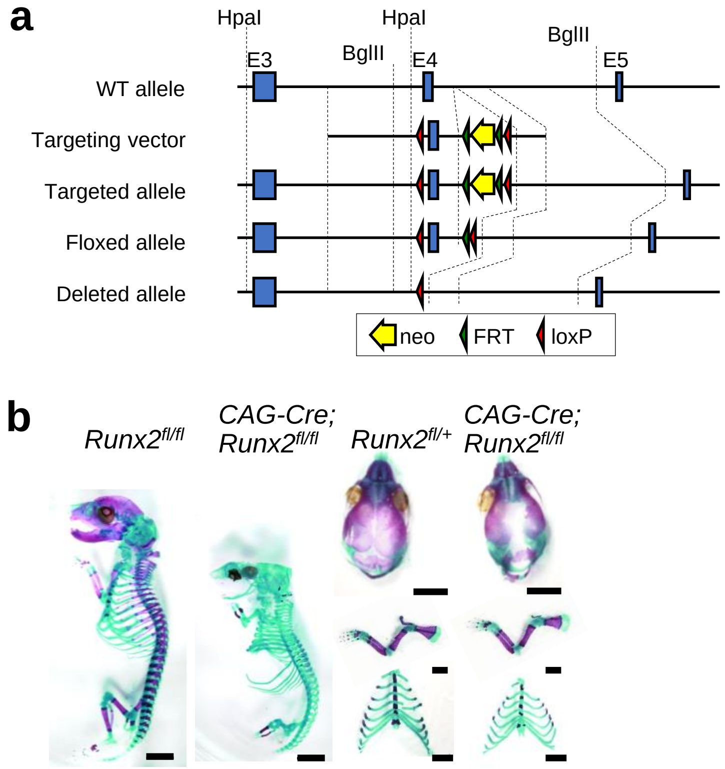

Supplementary Fig. 6 Generation and validation of *Runx2-flox* mice. **a** Generation of *Runx2*-flox mice. Diagram showing the Runx2 genomic locus with exons 4 and 5, *Runx2*-targeting vector including the neo-cassette (Targeting Vector), and recombination between homologous regions of the targeting vector and *Runx2* locus, leading to generation of the *Runx2*-targeted allele (Targeted Allele). The floxed *Runx2* allele was generated by flippase recombinase target (FRT)-mediated recombination of the *Runx2*-targeted allele (Floxed allele), and the Runx2-deleted allele was generated by Cre-mediated recombination of the floxed *Runx2* allele (Deleted allele). **b** Skeletal elements of E18.5 embryos of stained with Alcian blue (cartilage) and Alizarin red (mineralized tissue). Scale bars, 2 mm.

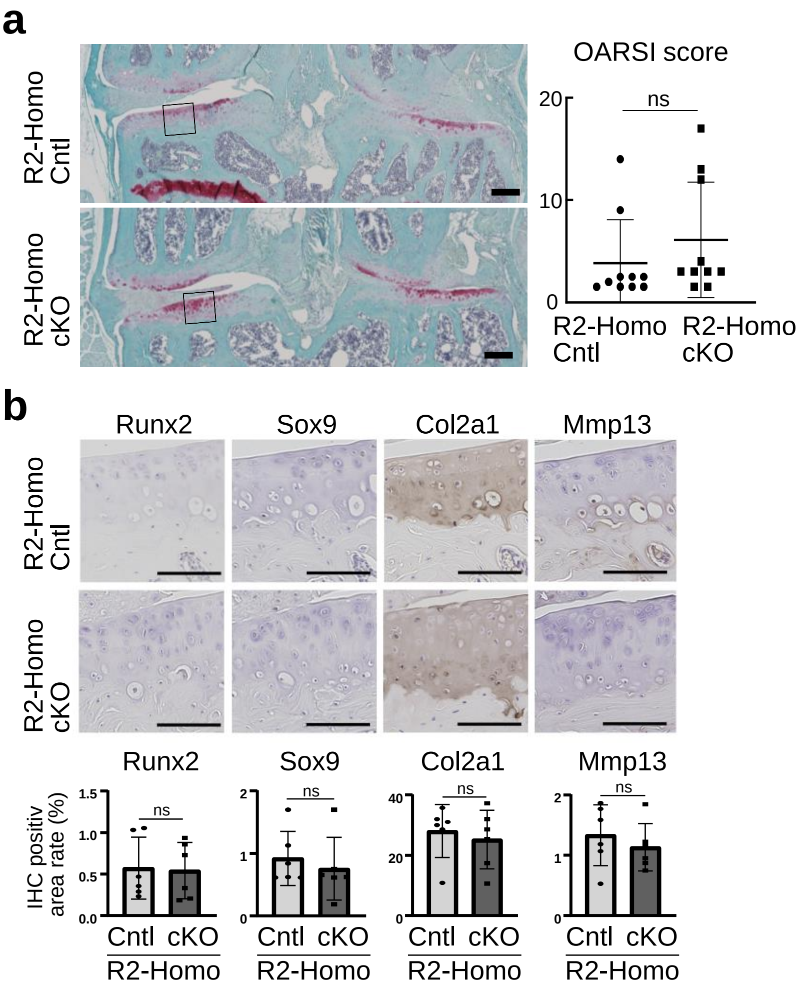

Supplementary Fig. 7 OA development of chondrocyte-specific Runx2-knockout mice with aging. **a** Representative Safranin O staining of 18-month-old homozygous *Runx2*$^{fl/fl}$ (R2-Homo Cntl) and *Col2a1-Cre*^{ERT2}*;Runx2*$^{fl/fl}$ (R2-Homo cKO) knee joints (aging model). OARSI scores are shown in the right panel. Data are expressed as mean ± S.D., n = 10 biologically independent animals. Scale bars, 200 μm. **b** Immunohistochemistry of Runx2, Sox9, Col2a1, and Mmp13 in 18-month-old R2-Homo Cntl and R2-Homo cKO knee joints. Six biologically independent animals which displayed OARSI scores close to the median for each group were analyzed. Scale bars, 100 μm. Inset boxes in Safranin O staining indicate regions of immunohistochemistry. All data are expressed as dot plots and mean ±S.D. ns, not significant; two-tailed Mann-Whitney U test.

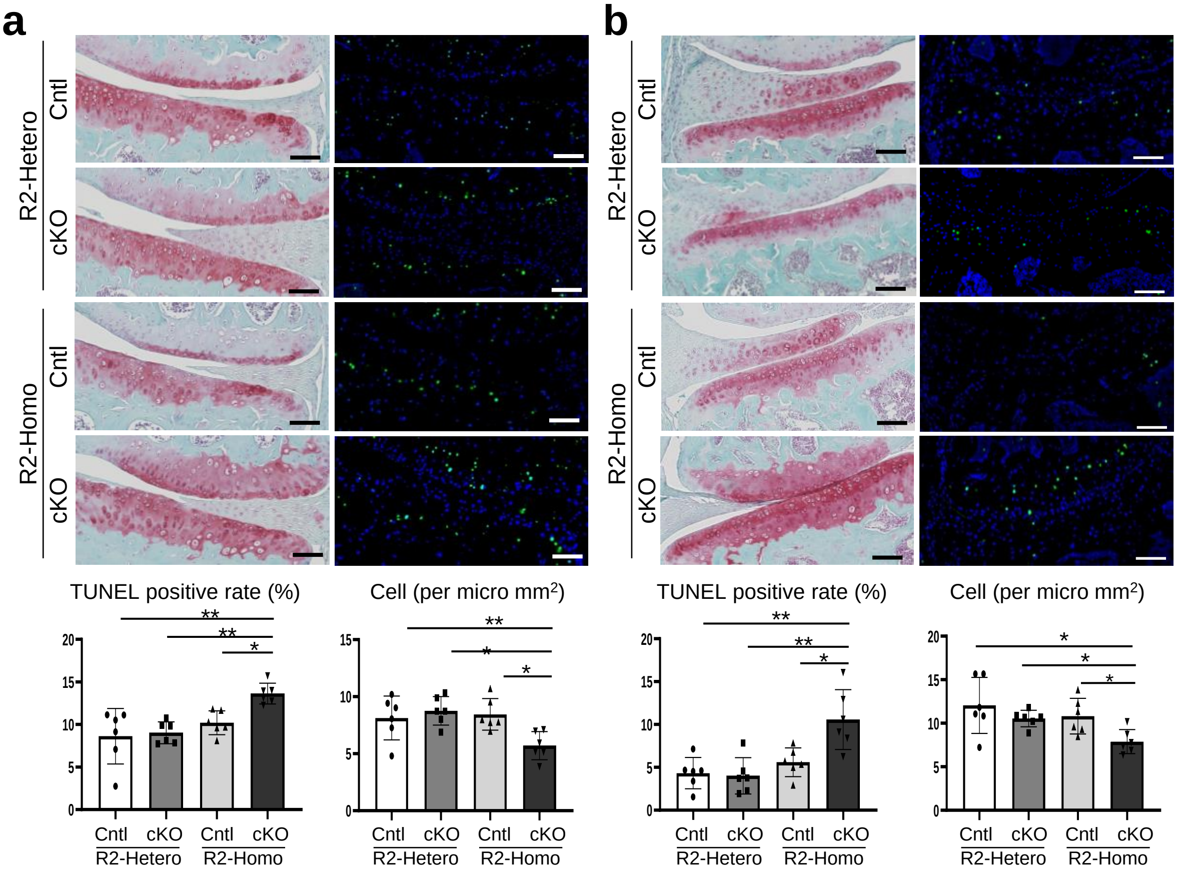

Supplementary Fig. 8 Chondrocyte apoptosis in articular cartilage of *Runx2$^{fl/+}$* (R2-Hetero Cntl), *Col2a1-Cre^{ERT2};Runx2$^{fl/+}$* (R2-Hetero cKO), *Runx2$^{fl/fl}$* (R2-Homo Cntl), and *Col2a1-Cre^{ERT2};Runx2$^{fl/fl}$* (R2-Homo cKO) mice. Safranin O and TUNEL staining of sham knee joints of medial model mice (**a**) and 8-week-old mice into which tamoxifen was injected at 7 weeks (**b**). Scale bars, 100 μm. Percentages of TUNEL-positive and DAPI-positive cells in the Safranin O-positive area are shown below. n = 6 biologically independent animals. All data are expressed as dot plots and mean ± S.D. *P < 0.05; **P < 0.01; one-way ANOVA test with Turkey's post hoc test.

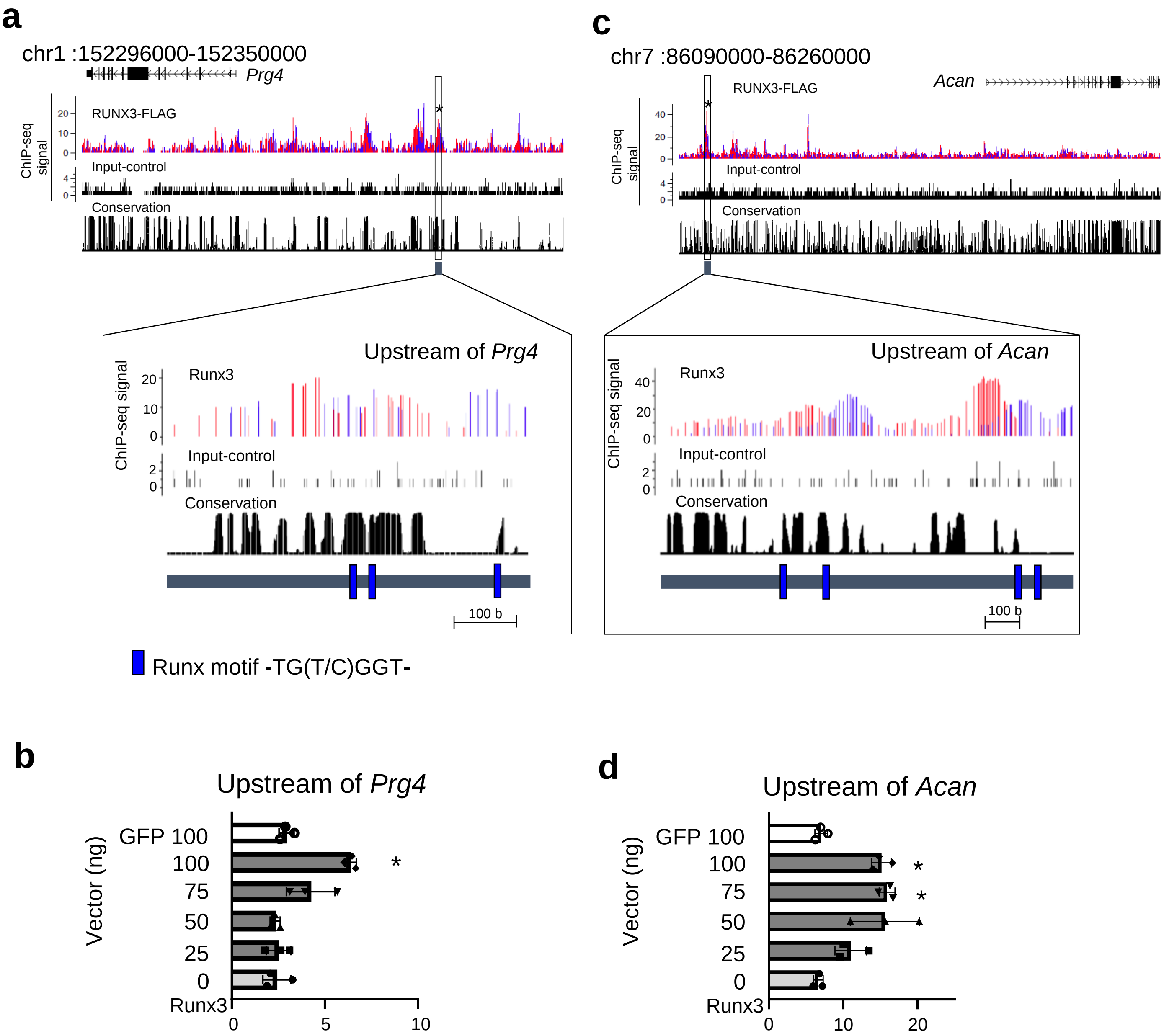

Supplementary Fig. 9 CisGenome browser screenshots showing Runx3 engagement, input control, and a conservation index around *Prg4* (**a**) and *Acan* (**c**) genes. Lower left and right panels indicate Chr1: 152337736−152338319 and Chr7: 86098728−86099908 in the mm9 database, respectively. Luciferase activities of *Prg4* **(b)** and *Acan* **(d)** enhancer regions in mouse chondrocytes transfected with various amounts of Runx3 or GFP control. n = 3 biologically independent experiments. Data shown as mean ± S.D. * indicates increased values, respectively, compared with control (white bars) with $P < 0.05$ in one-way ANOVA test with Turkey's post hoc test.

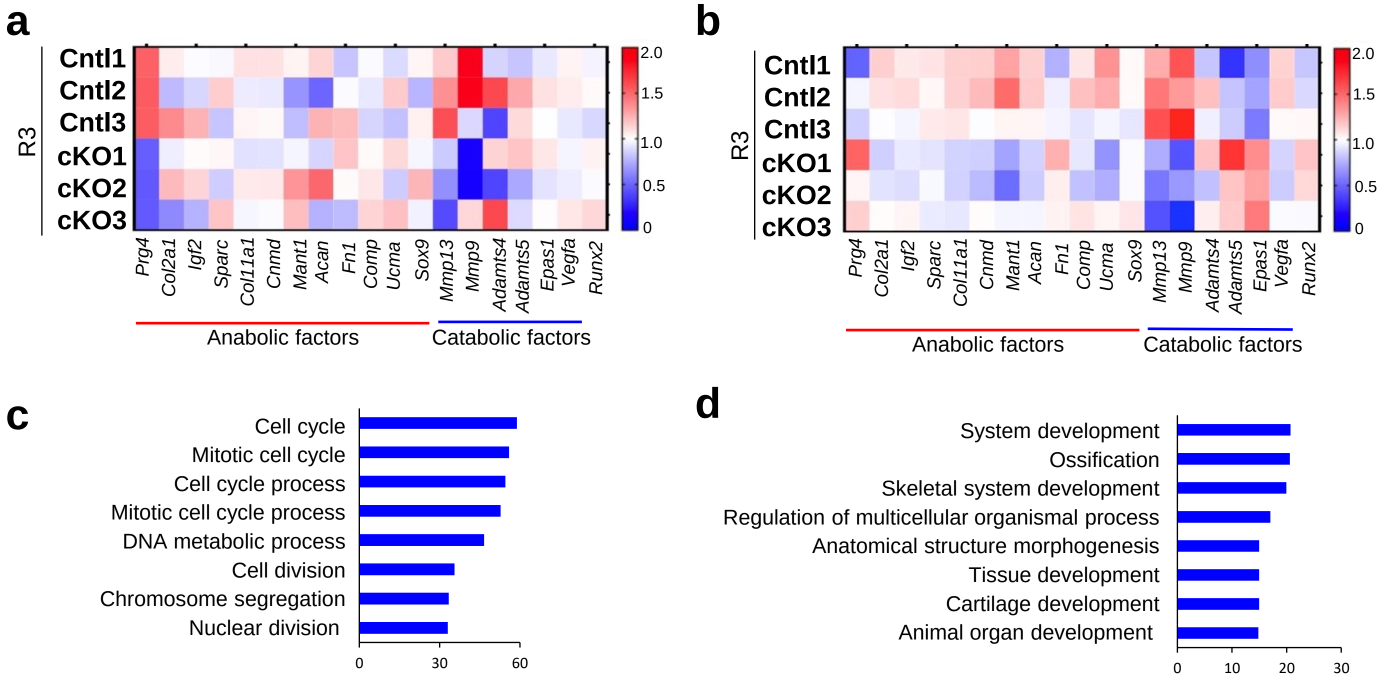

Supplementary Fig. 10 RNA-seq analyses of SFZ and DZ cells obtained from three individual *Runx3*$^{fl/fl}$ (R3-Cntl) and *Col2a1-Cre;Runx3*$^{fl/fl}$ (R3-cKO) littermate mice (n = 3). Heat maps showing extracellular matrix related genes, and articular cartilage catabolic factor related genes for SFZ (**a**) and DZ cells (**b**). Gene Ontology (GO) analyses in the top 2,000 downregulated genes in R3-cKO samples for SFZ (**c**) and DZ cells (**d**) showing top seven enriched GO terms.

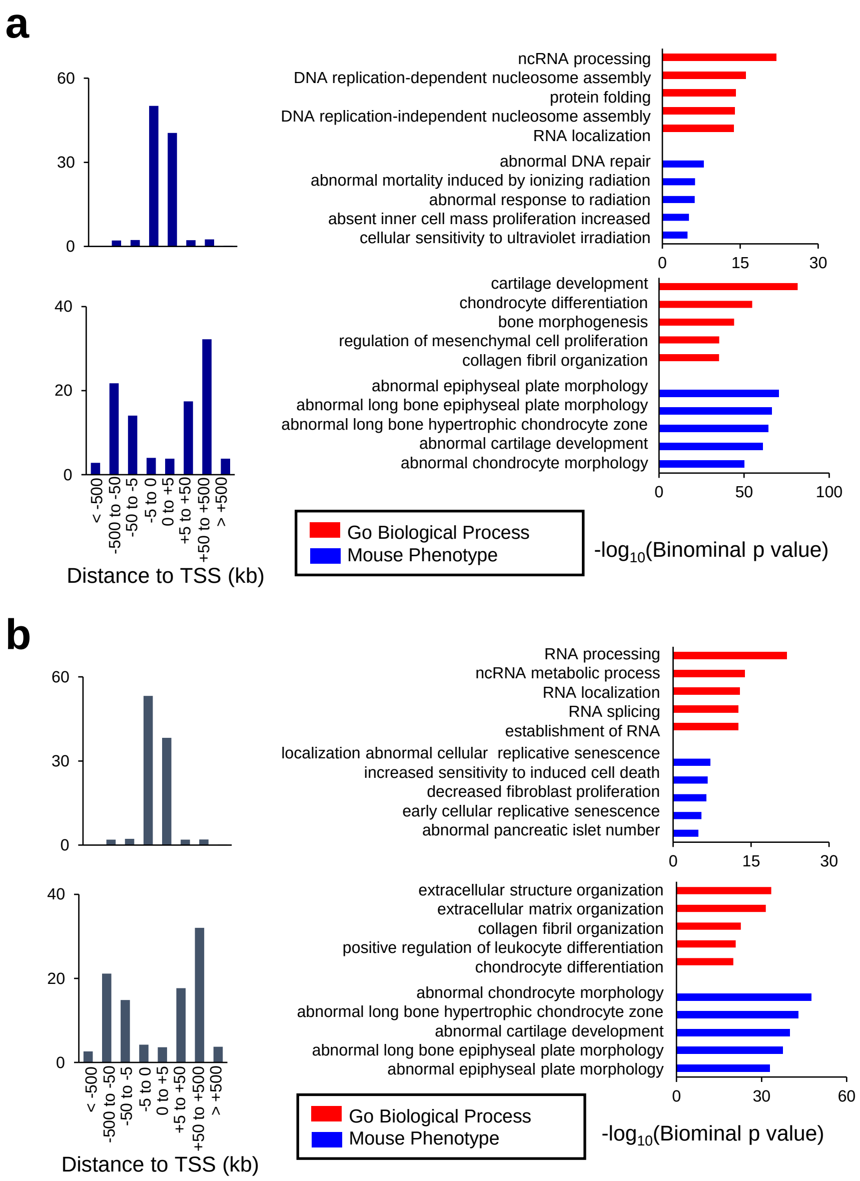

Supplementary Fig. 11 Genomic Regions Enrichment of Annotations Tool (GREAT), Gene Ontology (GO) analyses, and Mouse Genome Informatics (MGI) expression annotations excluding or limited to peaks with 500 bp of transcriptional start sites (TSSs) from Runx2-FLAG ChIP-seq data of primary chondrocytes. **a** Results of primary chondrocytes treated with vehicle. **b** Results of inflamed chondrocytes exposed to 1 ng per mL IL-1β.

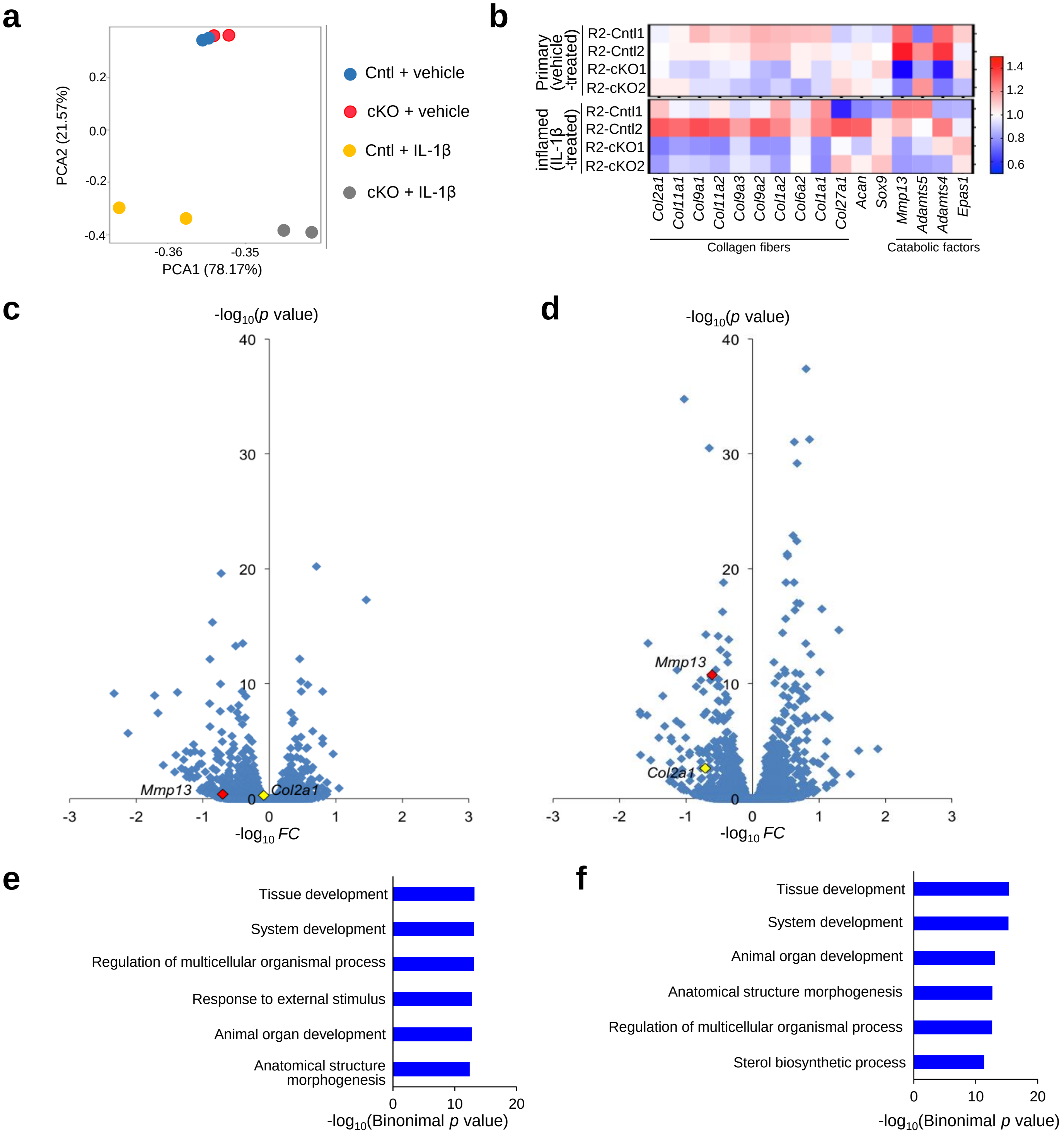

Supplementary Fig. 12 Downstream targets of Runx2 in chondrocytes under normal and inflammatory conditions determined by RNA-seq. **a** Principal component analysis plot. **b** Heat maps of collagen fiber-associated genes, *Acan*, *Sox9*, and catabolic factor-associated genes. **c, d** Volcano plots from RNA sequencing data comparing the positions of *Col2a1* and *Mmp13* in primary chondrocytes (**c**) and inflamed chondrocytes (**d**). **e, f** Genomic Regions Enrichment of Annotations Tool gene ontology analyses of the top 2,000 expressed genes in primary chondrocytes treated with vehicle (**e**) and inflamed chondrocytes exposed to 1 ng per mL IL-1β (**f**).

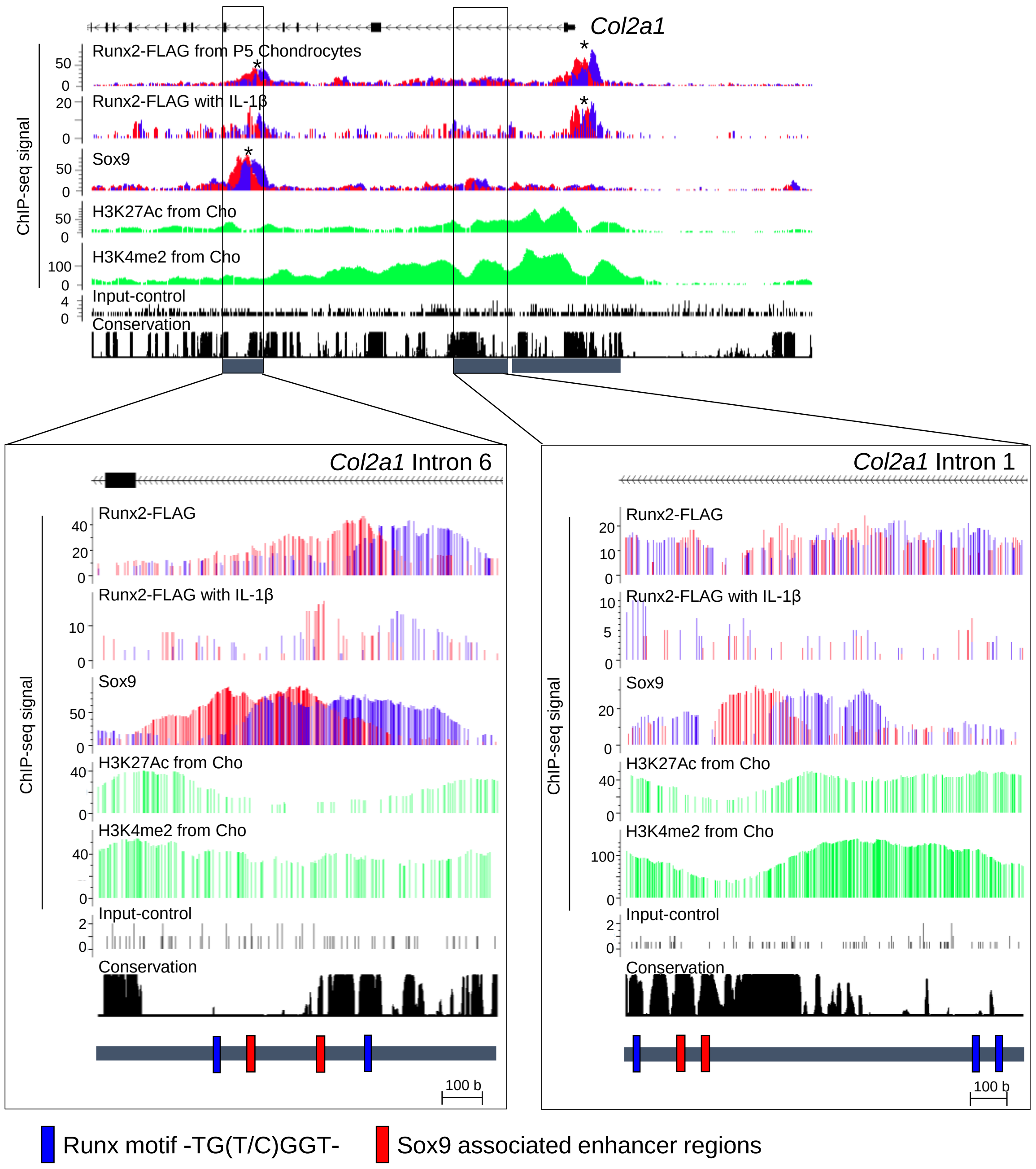

Supplementary Fig. 13 CisGenome browser views of Runx2 and reference engagement ChIP-seq around *Col2a1* (Chr15: 97825000–97840000). Lower panels show magnified views. Lower left and right panels indicate Chr15: 97827723-97828731 and Chr15: 97832509-97833631 in the mm9 database, respectively. ChIP-seq of Runx2-FLAG and Sox9 was performed by a paired-end method. Peak center positions are represented as red (data from forward reads) and blue (reverse reads) bars. Asterisks indicate peaks in ChIP-seq following peak calling criteria.

Supplementary Table 1. Top 10 down-regulated genes by Runx3 knockout in the SFZ cells, in order of FC value, evaluated by DESeq2 based on a model using the negative binomial distribution by two-tailed. FC, Fold Change.

				Average expression		
Rank	Gene Name	GeneID	Log2FC	R3-Cntl	R3-cKO	P value
1	*Prn*	111368	-2.273	15.897	3.289	< 0.001
2	*Mmp9*	17395	-1.523	158.309	55.094	0.001
3	*Tnnt2*	21956	-1.294	75.494	30.792	0.004
4	*Ccl2*	20296	-1.235	277.869	118.034	0.003
5	*Prg4*	96875	-1.153	1297.231	583.379	0.008
6	*Chrna1*	11435	-1.129	130.824	59.812	0.014
7	*Mylpf*	17907	-1.099	208.527	97.375	0.013
8	*Klhl30*	70788	-1.090	82.500	38.752	0.011
9	*Eln*	13717	-1.089	7882.319	3705.051	0.018
10	*Ibsp*	15891	-1.081	2259.332	1068.033	0.016

Supplementary Table 2. Top 10 down-regulated genes by Runx3 knockout in the DZ cells, in order of FC value, evaluated by DESeq2 based on a model using the negative binomial distribution by two-tailed. FC, Fold Change.

				Average expression		
Rank	Gene Name	GeneID	Log2FC	R3-Cntl	R3-cKO	P value
1	*C1qc*	12262	-1.885	12.788	3.462	< 0.001
2	*Zfp648*	100503355	-1.837	121.701	34.055	< 0.001
3	*Pthlh*	19227	-1.824	44.674	12.615	< 0.001
4	*Apoe*	11816	-1.772	320.277	93.774	< 0.001
5	*Tnfrsf19*	29820	-1.717	111.573	33.947	0.001
6	*Myo1f*	17916	-1.703	9.620	2.956	0.001
7	*Mpeg1*	17476	-1.701	31.592	9.715	0.001
8	*Alpl*	11647	-1.645	2480.344	793.254	< 0.001
9	*Col15a1*	12819	-1.614	1068.835	349.253	< 0.001
10	*Tnfrsf9*	21942	-1.592	27.470	9.112	< 0.001

Supplementary Table 3. Top 10 up-regulated genes by Runx3 knockout in the SFZ cells, , in order of FC value, evaluated by DESeq2 based on a model using the negative binomial distribution by two-tailed. FC, Fold Change.

				Average expression		
Rank	Gene Name	GeneID	Log2FC	R3-Cntl	R3-cKO	P value
1	*Fbxo15*	50764	1.131	4.764	10.437	0.013
2	*Lrrtm1*	74342	0.962	83.678	163.059	0.026
3	*Jph3*	57340	0.944	9.205	17.713	0.040
4	*Gm13083*	279185	0.921	2.690	5.092	0.044
5	*Penk*	18619	0.918	39282.223	74239.531	0.046
6	*NA*	NM_001305677	0.903	39.147	73.203	0.017
7	*Gsta4*	14860	0.890	218.037	404.136	0.040
8	*Cdkl4*	381113	0.876	4.695	8.619	0.056
9	*Cyp4f14*	64385	0.876	39.558	72.613	0.057
10	*Pm20d1*	212933	0.872	48.034	87.911	0.027

Supplementary Table 4. Top 10 up-regulated genes by Runx3 knockout in the DZ cells, in order of FC value, evaluated by DESeq2 based on a model using the negative binomial distribution by two-tailed. FC, Fold Change.

				Average expression		
Rank	Gene Name	GeneID	Log2FC	R3-Cntl	R3-cKO	P value
1	*Hoxc13*	15422	2.194	30.384	139.067	4.E-07
2	*Dsp*	109620	2.020	60.358	244.778	1.E-07
3	*Lce3c*	94060	1.885	2.154	7.957	2.E-04
4	*Car3*	12350	1.879	98.052	360.598	1.E-04
5	*Rab3b*	69908	1.758	205.651	695.508	3.E-04
6	*Crct1*	74175	1.726	3.783	12.517	0.001
7	*Serpine1*	18787	1.706	1017.046	3318.122	3.E-04
8	*Dlx2*	13392	1.699	48.422	157.176	1.E-05
9	*Slurp1*	57277	1.680	22.486	72.039	0.001
10	*Serpinb9b*	20706	1.675	10.446	33.347	0.001

Supplementary Table 5. Different expression gene lists of RNA sequencing in primary chondrocytes from *Runx2*$^{fl/fl}$ (R2-Homo Cntl) and *Runx2*$^{fl/f}$*;Col2a1-Cre*^{ERT2} (R2-Homo cKO) mice, in order of FC value, evaluated by DESeq2 based on a model using the negative binomial distribution by two-tailed.
FPKM, Fragments Per Kilobase of transcript per Million fragments Mapped; FC, Fold Change.

	Expression level (FPKM)			
Gene Symbol	R2-Homo Cntl	R2-Homo cKO	Log2 FC	P value
Tmem254b	1234.051	244.214	−2.337	7.280E−10
Mup18	29.671	6.787	−2.128	2.020E−06
Aoc3	222.588	67.298	−1.726	1.080E−09
Plin1	179.383	56.118	−1.677	3.650E−08
Gm3500	18.725	6.197	−1.595	1.266E−03
B3galt2	45.484	16.951	−1.424	4.828E−03
Adipoq	129.996	49.087	−1.405	1.629E−04
Bglap2	327.966	126.067	−1.379	5.640E−10
Car3	84.085	33.674	−1.32	2.366E−03
Il17b	116.841	47.88	−1.287	4.156E−04
Fabp4	733.023	304.746	−1.266	3.363E−03
Hhip	187.607	78.935	−1.249	7.640E−05
Hsd11b2	60.391	25.484	−1.245	1.256E−02
Wnt10b	67.869	29.581	−1.198	1.544E−02
Spink5	85.563	37.372	−1.195	8.184E−03
Gpd1	238.102	104.658	−1.186	1.022E−04
Olfml2a	113.961	50.739	−1.167	3.185E−03
Cd36	258.085	117.241	−1.138	1.940E−05
Lamc3	75.381	34.489	−1.128	1.664E−02
Hp	202.173	92.87	−1.122	7.590E−05
Ihh	128.499	59.727	−1.105	2.592E−03
Gper1	97.937	46.428	−1.077	1.119E−02
C3	201.53	97.416	−1.049	9.630E−04
Gli1	253.036	124.343	−1.025	1.118E−04
Podnl1	186.699	92.096	−1.02	9.524E−04

	Expression level (FPKM)			
Gene Symbol	R2-Homo Cntl	R2-Homo cKO	Log2 FC	P value
Ddx3y	21.352	1482.747	6.118	6.230E−79
Eif2s3y	11.411	605.925	5.731	5.450E−83
Kdm5d	9.557	424.667	5.474	3.230E−67
Uty	11.551	372.215	5.01	6.440E−59
NA	8.394	74.941	3.158	1.100E−15
Tcerg1l	5.633	16.355	1.538	2.317E−03
Esr1	229.908	632.026	1.459	4.980E−18
Pnp2	5.059	11.898	1.234	2.547E−02

Supplementary Table 6. Different expression gene list of RNA sequencing in inflamed chondrocytes (treated with 1 ng per mL IL-1β for 24 hours) from *Runx2*$^{fl/fl}$ (R2-Homo Cntl) and *Runx2*$^{fl/f}$*;Col2a1-Cre*^{ERT2} (R2-Homo cKO) mice, in order of FC value, evaluated by DESeq2 based on a model using the negative binomial distribution by two-tailed. FPKM, Fragments Per Kilobase of transcript per Million fragments Mapped; FC, Fold Change.

	Expression level (FPKM)			
Gene Symbol	R2-Homo Cntl	R2-Homo cKO	Log2 FC	P value
Mylpf	160.668	49.620	−1.695	2.840E−08
Myh1	56.546	17.597	−1.684	1.654E−04
Myh8	145.108	45.163	−1.684	5.030E−08
Actc1	165.294	54.993	−1.588	5.580E−08
Ibsp	367.421	123.671	−1.571	3.070E−14
Ttn	66.201	22.936	−1.529	4.641E−04
Sp7	154.509	58.208	−1.408	5.260E−06
Podnl1	291.177	114.184	−1.351	1.180E−09
Bglap	205.567	82.190	−1.323	5.180E−07
Atp2a1	46.853	19.733	−1.248	3.180E−02
Des	108.582	46.054	−1.237	7.607E−04
Myog	42.461	18.123	−1.228	3.025E−02
Acta1	231.711	99.026	−1.226	5.090E−06
Neb	57.484	24.605	−1.224	1.478E−02
Myh3	202.111	87.078	−1.215	1.060E−05
Smpd3	1754.054	789.284	−1.152	4.429E−03
Tmem119	298.302	134.934	−1.145	1.840E−07
Alpl	518.483	236.375	−1.133	6.690E−12
Mfap5	82.824	37.776	−1.133	9.406E−03
Wnt10b	202.773	94.877	−1.096	5.020E−05
Bglap2	362.647	172.052	−1.076	3.670E−07
Thy1	181.539	86.608	−1.068	3.491E−04
Myl1	132.910	63.682	−1.061	2.953E−03
Gpx3	4298.849	2110.731	−1.026	1.700E−35
Ddx3y	1857.833	925.398	−1.005	5.180E−06

	Expression level (FPKM)			
Gene Symbol	R2-Homo Cntl	R2-Homo cKO	Log2 FC	P value
NM_001305819	7.208	51.933	2.849	8.93E−11
Raet1b	6.099	25.708	2.076	1.54E−05
Ifit1	12.508	46.196	1.885	4.83E−05
Ces2e	7.871	25.444	1.693	1.13E−03
Ano3	28.708	86.801	1.596	6.95E−05
Gm14430	88.864	246.747	1.473	7.63E−03
Capn11	6.223	16.153	1.376	1.76E−02
Mrgprf	222.209	546.158	1.297	2.20E−15
Stfa3	26.378	62.782	1.251	8.69E−03
Itga2	28.302	66.838	1.240	7.50E−03
S100a8	15.676	36.606	1.224	3.99E−02
H60a	68.454	157.252	1.200	4.47E−02
Sncg	40.474	92.550	1.193	3.67E−03
Chdh	138.242	307.787	1.155	9.84E−08
Serpina3n	2012.897	4354.166	1.113	1.12E−05
Mgmt	84.358	179.775	1.092	2.14E−04
Esr1	168.661	359.174	1.091	5.10E−08
Ptgs1	474.049	975.514	1.041	3.21E−17
Notch3	81.955	165.771	1.016	1.00E−03
Dglucy	319.079	644.004	1.013	9.77E−12

Supplementary Table 7. Primers used for genotyping.

Runx2-flox	F	aacatacaaactgtcttccctcaac
	R	caggtcttgagctaaaagcagaagg
Cre	F	gcatttctggggattgctta
	R	cccggcaaaacaggtagtta
Prg4-Cre^{ERT2}	Common F	tgactatctcaggaattcaagctg
	Wild type R	cgcttttgacgctgaagttac
	Mutant R	gaacttcagggtcagcttgc
Runx3-flox	F	tatccctctctgggccttct
	R	ggaaactgagtccagccaag
Runx2-FLAG	tag-F	gcttgtcatcgtcatccttgtagtc
	F	ttgcaagatcatgactagggatt
	R	ccccaactgttttgaattctagc

Supplementary Table 8. Primers used for qRT-PCR.

Gene Symbol	Species		Sequence
Gapdh	mouse	F	tgcaccaccaactgcttagc
		R	ggatgcagggatgatgttct
Runx3	mouse	F	tcacaatcaccgtgttcacc
		R	ctgggtgtgcttggtgttac
Runx2	mouse	F	gactgtggttaccgtcatggc
		R	acttggtttttcataacagcgga
Runx1	mouse	F	ccagcctctctgcagaactt
		R	caggtaggtgtggtagcgaga
Prg4	mouse	F	tccattcagaccaccatctcc
		R	gagcagccactatcttccctgt
Acan	mouse	F	ccaaaccagcctgacaactt
		R	tctagcatgctccaccactg
Col2a1	mouse	F	gccaagacctgaactctgc
		R	gccatagctgaagtggaagc
Sox9	mouse	F	cgactacgctgaccatcaga
		R	agactggttgttcccagtgc
Mmp9	mouse	F	tgaatcagctggcttttgtg
		R	gtggatagctcggtggtgtt
Mmp13	mouse	F	aggccttcagaaaagccttc
		R	tccttggagtgatccagacc
Vegfa	mouse	F	caggctgctgtaacgatgaa
		R	tttcttgcgctttcgttttt
Hif2a	mouse	F	tgagttggctcatgagttgc
		R	ctcacggatctcctcatggt

Supplementary Table 9. Locations of the cloned sequences for luciferase reporter vectors.

Reporter	Colned sequence (MGSCv37/mm9)	Length
Col2a1 Intron6	Chr15:97827723-97828731	1,009 bp
Col2a1 Intron1	Chr15:97832509-97833631	1,123 bp
Col2a1 Around TSS	Chr15:97833508-97835758	2,251 bp
Mmp13 Upstream of TSS	Chr9:7271588-7272547	960 bp
Prg4 Upstream	Chr1:152337736-152338319	584 bp
Acan Upstream	Chr7:86098728-86099908	1,181 bp
